# Evaluation of a novel liquid stabilised peste des petits ruminants vaccine: Safety and immunogenic efficacy in sheep and goats in the field in Jordan

**DOI:** 10.1016/j.jvacx.2023.100363

**Published:** 2023-08-04

**Authors:** Fraser Crofts, Ahmad Al-Majali, David Gerring, Simon Gubbins, Hayley Hicks, Dana Campbell, Steve Wilson, Lizzie Chesang, Kristin Stuke, Claudia Cordel, Satya Parida, Carrie Batten

**Affiliations:** a The Pirbright Institute, Ash Road, Pirbright, Surrey GU24 0NF, United Kingdom; b Faculty of Veterinary Medicine, Jordan University of Science and Technology, Irbid 22110, Jordan; c Subregional Office for the Gulf Cooperation Council States and Yemen, Food and Agriculture Organization of the United Nations (FAO), Abu Dhabi 62072, United Arab Emirates; d Dana Campbell Consultants Ltd, 15 Justice Park, Oxton, Lauderdale TD2 6NZ, United Kingdom; e GALVmed, International Livestock Research Institute (ILRI), Swing One, Naivasha Road, Nairobi, Kenya; f Arecor Therapeutics PLC, Chesterford Research Park, Little Chesterford, Saffron Walden CB10 1XL, United Kingdom; g Food and Agriculture Organization of the United Nations (FAO), Viale delle Terme di Caracalla, 00153 Rome, Italy; h GALVmed, Doherty Building, Pentlands Science Park, Bush Loan, Edinburgh EH26 0PZ, United Kingdom

**Keywords:** PPR vaccine, Sheep, Goat, Liquid stabilised vaccine, Lyophilised vaccine

## Abstract

A novel liquid stabiliser was tested with the Nigeria 75/1 Peste des Petit Ruminants (PPR) vaccine over two field studies carried out in sheep and goats. PPR seronegative sheep and goats were selected from farms surrounding Amman, Jordan and were vaccinated with either a stabilised liquid PPR vaccine that had been formulated 3 months prior to use and stored at 2–8 °C or a reconstituted lyophilised PPRV vaccine reconstituted on the day of vaccination. Sera were taken immediately before vaccination and at approximately 1.5, 3 and 6 months following vaccination, then subsequently tested using IDVet ID Screen® PPR competition ELISA and Serum Neutralisation tests to determine the presence of PPRV anti-N antibodies and neutralising antibodies, respectively. It was observed that the liquid-stabilised vaccine was able to provide comparable antibody responses in both species to those induced by the lyophilized vaccine. The ability to store liquid stabilised PPRV vaccine for field use would positively impact PPRV eradication efforts.

## Highlights

• PPRV Nigeria 75/1 vaccine provides at least 52 weeks of shelf-life at 4 °C when formulated with a liquid stabiliser.
• Liquid stabilised vaccine induces detectable PPRV antibodies for at least 6 months in sheep and goats.
• The use of liquid stabilised vaccine produces no observable loss in serum antibody titre compared to that induced by freeze-dried vaccine.

## Introduction

Peste des Petits Ruminants (PPR) is a transboundary disease with high morbidity and mortality in ovine and caprine species [1]. The disease is caused by Peste des Petits Ruminants virus (PPRV), a small ruminant Morbillivirus, which shares a single genus with other viruses capable of causing notable diseases including canine distemper [2], human measles [3] and the recently eradicated cattle disease rinderpest [4]. PPR continues to result in major agricultural financial costs in affected communities, with an annual attributable economic loss of 1.4–2.1 billion USD [5]. Following the successful eradication of rinderpest, The Food and Agriculture Organization of the United Nations (FAO) and the World Organisation of Animal Health (WOAH) have now resolved to eradicate PPR by 2030, a goal which would not only eliminate a major threat to the wellbeing of sheep and goats, but additionally have positive global economic impacts [6].

A lesson learned from the rinderpest eradication campaign was that such a strategy necessitates a high degree of logistical coordination regarding vaccine roll-out, catered specifically to the needs of affected areas [7]. A major consideration in this matter regards maintenance of the cold-chain due to live-attenuated PPR vaccines being highly thermolabile [8] and the endemic regions of PPR fall predominantly within the northern tropics and equatorial climates [9]. Furthermore, the nature of animal husbandry in affected nations requires the shipment of vaccines to isolated towns and villages, far removed from major distribution hubs [10]. To mitigate the resource requirements and consequences of failure of the cold-chain, efforts have been made to develop liquid vaccine stabilisers that allow for ambient storage and delivery of vaccine as an alternative to lyophilization procedures. Further benefits of this would be the availability of correct pre-prepared dosages, reducing vaccine administration errors resulting from improper reconstitution, in addition to financial benefits of reducing capital expenditure and on-going process costs of lyophilisation.

In this study, we assess whether a liquid vaccine stabiliser provides substantial preservation of vaccine virus titre in long-term refrigerated storage. Furthermore, we compare the antibody responses induced by a liquid-stabilised vaccine with those induced by a traditional reconstituted lyophilized vaccine formulation within a population of sheep and, separately, goats. The results of these studies enable the assessment of the liquid stabilised vaccines efficacy and help in evaluating its potential use to support the ongoing PPR eradication efforts.

## Materials and methods

### Liquid stabaliser - Arecor therapeutics PLC (Cambridge, UK)

The stabiliser is a clear and colourless aqueous liquid, with viscosity and pH comparable to water. The stabiliser contains a mixture of chemical stabilisers that are accredited as GRAS (generally recognised as safe) by the Food and drug administration. The liquid stabiliser contains a displaced buffering system and is isotonic.

### Virus titration

The liquid stabiliser was used was used to dilute a known titre of PPRV Nigeria 75/1 (Nig75/1) at a 1 in 10 vol ratio. Aliquots (and a virus only control) were stored at 4 °C for a period of 52 weeks, with virus titrations to determine stability and viability performed at timepoints of 0, 2, 12, 26, 38 and 52 weeks post-formulation[18].

### Field study

Live-attenuated Nig75/1 vaccine (Pestevac, Batch No. 231120, Jordan Bio-industries Center, Jordan) was used throughout the study. The vaccine was formulated with a proprietary liquid stabiliser supplied by Arecor, UK, three months prior to vaccination with a recorded titre of 1 × 10^3.1^ TCID_50_ and stored between 2 °C − 8 °C, with a recorded titre at D0 of 1 × 10^2.9^ TCID_50_. Lyophilized vaccine was stored between 2 °C − 8 °C and was reconstituted to 1 × 10^3.0^ TCID_50_ on the day of vaccination in sterile Phosphate Buffered Saline (PBS) based diluent.

Sheep belonging to the Awassi breed were sourced from five farms surrounding the city of Amman, Jordan. A total of 160 sheep aged 5–8 months were selected and screened to confirm they were seronegative for PPRV. The sheep were allocated to groups: 75 sheep within Group 1, “Liquid PPRV Vaccine” (Arecor Liquid Stabiliser + Nig75/1), 75 sheep within Group 2, “Freeze Dried Vaccine” (Lyophilized Nig75/1) and 10 sheep within Group 3 “Control” (Arecor Liquid Stabiliser only). 110 Baladi-Shami crossbreed goats were sourced from 2 farms from Amman district and screened to confirm they were seronegative for PPRV. The goats were allocated to the same three groups in numbers of 50 goats to Group 1 and Group 2, and 10 goats to Group 3. Group sizes were calculated to ensure that the study would be able to show the performance of the liquid PPRV vaccine was equivalent to that of the freeze dried vaccine.

The vaccines were administered 1 mL subcutaneously to the left side of the neck. Animals were observed and rectal temperatures and adverse health effects were recorded by veterinary personnel for 7 days in addition to weekly check-ins through to the completion of the study. Following day 0, blood samples (pre-vaccination bleed) were drawn by venepuncture of the jugular vein at intervals of 1.5 months (45/46dpv sheep, 36dpv goats), 3 months (92dpv sheep, 82dpv goats) and 6 months (181/182dpv sheep, 172dpv goats) post-vaccination. Serum was separated and heat-inactivated at 56 °C for 30 min, prior to refrigerated shipment to The Pirbright Institute, UK. Serum samples were stored refrigerated for the duration of the study.

### Laboratory analysis

Serum samples were screened for the presence of PPRV antibodies using the IDvet ID Screen® PPR Competition ELISA (ID VET, France) following the manufacturer’s instructions and for neutralising antibodies by Serum Neutralisation Tests (SNTs) [17].

### Statistical methods

Viral titres with and without the addition of the liquid stabiliser were compared using linear regression, with log TCID_50_ as the response variable and week and treatment (control or stabilised) as explanatory variables. Model selection proceeded by stepwise deletion of non-significant (*P* > 0.05) terms (as judged by *F*-tests), starting from a model including both explanatory variables and an interaction between them.

Because of non-equal variances and non-normality of residuals, the ELISA S/N ratios and serum neutralisation titres for animals vaccinated using the liquid or freeze-dried formulations were compared at each time point using Wilcoxon rank-sum tests (two-sided, unpaired) with a threshold for significance of *P* = 0.05. Serum neutralisation test titres reported as >256 were arbitrarily set to 512 for analysis. In addition, the numbers of positive animals in each treatment group (i.e. those with titre >10 and those with an S/N ratio ≤50%) at each time point were compared using *χ*^2^ tests.

All analyses were implemented in R (version 4.1.3) [19].

## Results

### Virus titration of PPRV Nig75/1 in liquid stabiliser

**Fig. 1:**
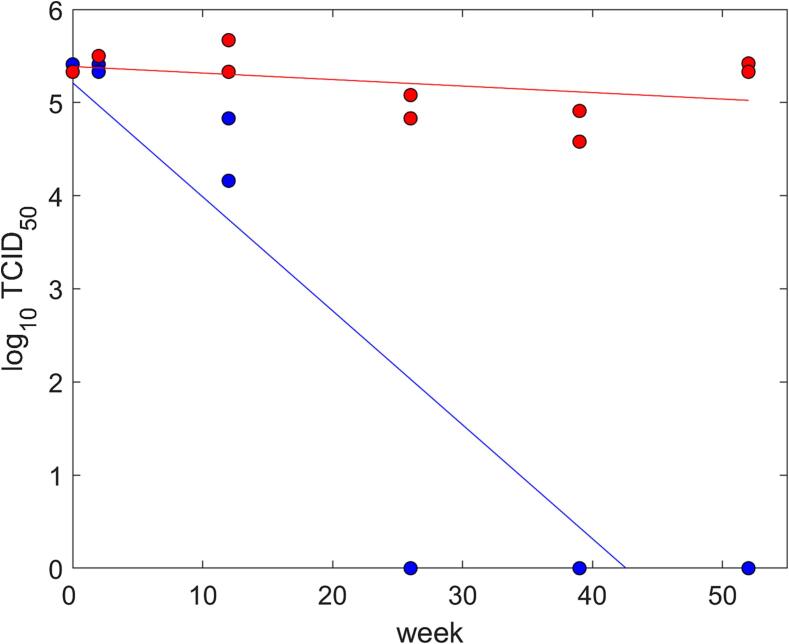
Changes in viral titre (log_10_ TCID_50_) over time for peste des petits ruminants virus (PPRV) Nig75/1 in liquid stabiliser at 4 °C storage over time compared to PPRV Nig75/1 control. Observed titres are shown as circles with colour indicating treatment group: virus with stabiliser (red) and virus alone (blue). The lines are those fitted to the data by linear regression. (For interpretation of the references to colour in this figure legend, the reader is referred to the web version of this article.)

Changes in titre over time differed significantly (*P* < 0.001) between the virus with stabiliser and the virus alone (Fig. 1). The titre of the virus with stabiliser did not change significantly (*P* = 0.15) (estimated slope = -0.007; 95% confidence interval (CI): −0.017 to 0.003), while the titre of the virus control decreased significantly (*P* < 0.001) over time (estimated slope = -0.12; 95% CI: −0.16 to −0.08).

### Vaccine safety

Throughout the course of the study, animals were observed by veterinary personnel in order to determine if there were any localised or systemic adverse effects to the health of the animals that could be attributed to the administered formulation. No such adverse effects were recorded.

### Enzyme-linked immunosorbent assay

**Fig. 2:**
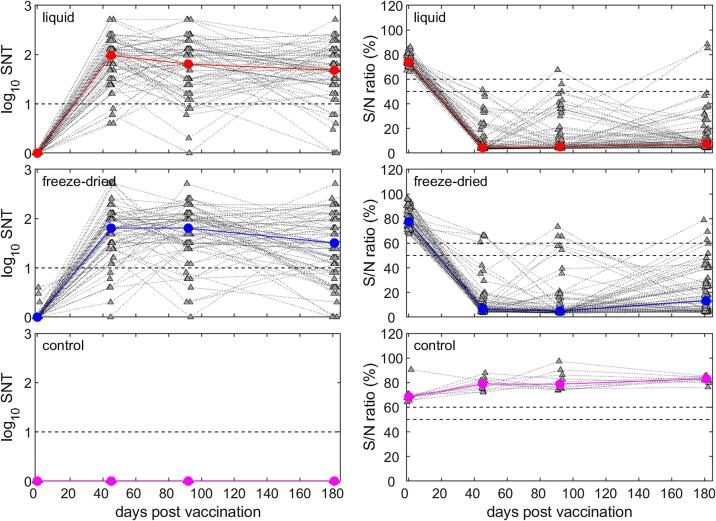
Antibody responses in sheep after vaccination against peste des petits ruminant virus as measured by serum neutralisation test (SNT; left-hand column) and ELISA S/N ratio (%; right-hand column). Sheep were vaccinated with a liquid-stabilised vaccine (top row), a reconstituted freeze-dried vaccine (middle row) or the stabiliser alone (control; bottom row). In each panel the coloured lines and circles show the median response for each treatment group, while the grey triangles and dotted lines show the responses for individual sheep. The black dashed lines indicate the thresholds for each assay; response for an animal to be considered positive for SNT (>10) and the inconclusive band for the ELISA S/N ratio (50–60%).

The absence of PPRV antibodies (confirmed by ELISA) in all enrolled sheep at D0 verified that all animals had no exposure to PPRV prior to the commencement of the study (Fig. 2). At the first sampling, 45/46dpv the majority of animals in both experimental groups were positive for PPRV antibodies. As the study progressed to 92dpv and 181/182dpv the degree of participating animals positive for PPRV antibodies remained consistent, with a deviation of only one animal in either group. Throughout the study, animals in the control group remained negative for PPRV antibodies (Fig. 2).

There were significant differences in median ELISA S/N percentages between sheep receiving liquid and freeze-dried formulations of the vaccine at D0 (*P* < 0.001; liquid (73.9%) < freeze-dried (77.4%)) and 45/46dpv (*P* = 0.003; liquid (4.5) < freeze-dried (6.6)). However, the proportion of positive sheep did not differ significantly between the treatment groups at any time point (*P* > 0.28). Furthermore, the statistical difference at D0 is irrelevant due to all variation being within degrees of negative response in the assay, rather than variation in genuine signal.

At D0, all goats within the Liquid PPRV Vaccine group tested negative for the presence of PPRV antibodies (Fig. 2). Within the Freeze-dried Vaccine group four goats were withdrawn from the study following confirmation of positive ELISA results, resulting in a total of 46 negative goats within the group for the remainder of the study. Across the following time points, all animals in each of the experimental groups tested positive for PPR antibodies. Throughout the study, animals in the control group remained negative for PPRV antibodies (Fig. 2).

There were significant differences in median ELISA S/N ratios between goats receiving liquid and freeze-dried formulations of the vaccine at 36dpv (*P* < 0.001; liquid (5.0%) < freeze-dried (7.3%)) and 82dpv (*P* = 0.03; liquid (6.1%) > freeze-dried (5.3%), but not at 172dpv (*P* = 0.78). This indicates a higher initial reaction in the liquid PPRV vaccine group followed by a more pronounced mid-term reaction in the freeze-dried group, before both groups equalise relative to each other by the end of the study.

### Serum neutralisation test

**Fig. 3:**
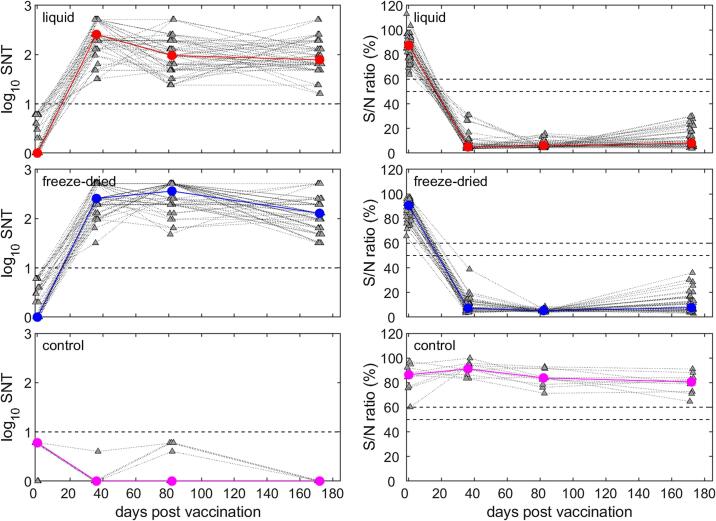
Antibody responses in goats after vaccination against peste des petits ruminant virus as measured by serum neutralisation test (SNT; left-hand column) and ELISA S/N ratio (%; right-hand column). Goats were vaccinated with a liquid-stabilised vaccine (top row), a reconstituted freeze-dried vaccine (middle row) or the stabiliser alone (control; bottom row). In each panel the coloured lines and circles show the median response for each treatment group, while the grey triangles and dotted lines show the responses for individual goats. The black dashed lines indicate the thresholds for each assay; response for an animal to be considered positive for SNT (>10) and the inconclusive band for the ELISA S/N ratio (50–60%).

All sheep serum samples at D0 were negative for neutralising antibodies against PPRV (Fig. 3). At 45dpv neutralising antibodies were detected in serum samples from animals across the two experimental groups. By the end of the study, the number of positive sheep in each experimental group had diverged, with 66 positive sheep from the Liquid PPRV Vaccine compared to 56 in the Freeze-dried Vaccine group, and 7 negative sheep in liquid vaccine compared to 14 in the freeze-dried group. The control sheep’s’ seronegative status was consistent throughout the study (Fig. 3).

All goat serum samples at D0 were negative for neutralising antibodies against PPRV (Fig. 3). At the remaining timepoints all goats in both experimental groups were positive for the presence of neutralising antibodies. Within the control group all goats remained negative (Fig. 3).

In sheep, there were significant differences in median SNT titre between treatment groups at 45/46dpv (*P* = 0.020; liquid (96) > freeze-dried (64)) and 181/182dpv (*P* = 0.007; liquid (48) > freeze-dried (32)). The proportion of positive sheep was significantly different at 181/182dpv (*P* = 0.02) and as such 181/182dpv is the only time point at which there is significant deviation in both quantitative and qualitative serum neutralisation titre metrics. 45/46dpv was the only timepoint at which both the ELISA S/N ratio and the SNT data were consistently significantly different with the liquid vaccine formulation provoking the stronger response; 181/182dpv has numerical consistency due to an appropriate lower S/N percentage in the liquid vaccine formulation (7.4%<13.3%) yet does not meet the threshold of statistically significant difference (*P* = 0.07).

Comparatively the statistical differences in numerical SNT titres in goats are more minor than in sheep. There was a significant difference in median SNT titre between treatment groups at 82dpv (*P* < 0.001; liquid vaccine (96) < freeze-dried (3 8 4)), but not at any other time point (*P* > 0.15). This in conjunction with the ELISA data highlights 82dpv as the point of convergence between the two formulations with the freeze-dried formulation eliciting a more pronounced effect at this timepoint. However, by the end of the study both groups are statistically similar and show total antibody presence.

## Discussion

These findings present a positive indication for a use-case of liquid vaccine stabiliser during the eradication campaign against PPR. The formulation as designed by Arecor has a demonstrable impact on limiting viral titre loss of the Nig75/1 vaccine in direct comparison to an equivalent reconstituted dosage form of the vaccine. Nig75/1 was shown *in vitro* to have a shelf-life limited to between 12 and 26 weeks at standard refrigeration temperatures, while the liquid stabiliser enabled the virus to maintain its original titre for a full 52 weeks (data not shown). The use of this liquid stabiliser to reconstitute Nig75/1 vaccine would greatly increase the utility of vaccine produced for an eradication campaign, not least because 52 weeks of guaranteed storage would allow shipment of vaccine to a location to be able to cover multiple seasons of seasonal PPR exposure or accommodate the long-term staggered vaccination of a growing herd. There would additionally be a financial benefit due to the removal of costly large-scale lyophilisation costs.

With observation *in vivo* of the use of the liquid stabilised vaccine in both sheep and goats within agricultural conditions, there is a clear indication that a liquid-stabilised Nig75/1 vaccine formulation has promise in a practical application. Both the ELISA and Serum Neutralising Test data clearly demonstrated that the liquid-stabilised vaccine formulation, following three months of storage in liquid form, elicited a serological immune response in sheep and goat populations. Animals were shown to seroconvert to PPRV and develop a neutralising immune response indicative of protection from PPRV infection. In fact, there is an indication that the liquid stabilised formulation is potentially providing sheep with an antibody response with a greater degree of longevity than the traditional lyophilized formulation; the decline in neutralising antibody titre at 182 days in the lyophilized group may be a sign of an accelerated loss of potential protection in the animals, despite the liquid formulation having been stored for three months prior to commencement of the study. Furthermore, in goats it has been shown that both formulations illicit neutralising antibodies over a 6-month period. It should be noted that sheep and goats naturally produce differing responses to PPRV infection including variance in viral load and antibody titre [16]. Further investigation into the longer-term immune response in animals up to a year, to observe any possible decline of neutralising antibodies and to determine if the immune response could potentially cover the seasonal variances that influence PRPV [11] would be beneficial. Additionally further study into the use of this liquid-stabilised vaccine in a variety of geographic areas would provide greater insight into the efficacy of the liquid-stabilised vaccine beyond the specific environmental conditions of Jordan, particularly in terms of variance based on meteorological factors [12], humidity [13] and current endemic status of the test region [14]. It could also be warranted to investigate how PPR vaccine stored in a liquid stabiliser is affected by sub-zero storage temperatures caused by shipment to remote mountainous regions, as has been previously described in the western provinces of China [15].

## Declaration of Competing Interest

The authors declare that they have no known competing financial interests or personal relationships that could have appeared to influence the work reported in this paper.

## Data Availability

Data will be made available on request.
